# The variability of baroreflex sensitivity in juvenile, spontaneously hypertensive rats

**DOI:** 10.5830/CVJA-2010-007

**Published:** 2011

**Authors:** Vitor E Valenti, Celso Ferreira, Luiz Carlos De Abreu, Eduardo Colombari, Monica A Sato

**Affiliations:** Department of Medicine, Cardiology Division, UNIFESP, São Paulo, Brazil; Department of Medicine, Cardiology Division, UNIFESP, São Paulo, Brazil; Cardiology Division, School of Medicine of ABC, Santo André, Brazil; Department of Public Health, University of São Paulo, Brazil; Department of Morphology and Physiology, School of Medicine of ABC, Santo André, Brazil; Department of Morphology and Physiology, School of Medicine of ABC, Santo André, Brazil

**Keywords:** baroreflex, rats, inbred SHR, sympathetic nervous system, parasympathetic nervous system, autonomic nervous system

## Abstract

In this study the baroreflex sensitivity of conscious, juvenile, spontaneously hypertensive rats (SHRs) was compared. The study population consisted of 19 eight-week-old male SHRs. The baroreflex sensitivity was quantified as the derivative of the variation in heart rate (HR) and the variation of mean arterial pressure (baroreflex sensitivity = ΔHR/ΔMAP).

MAP was manipulated with sodium nitroprusside (SNP) and phenylephrine (PHE), administered via an inserted cannula in the right femoral vein. The SHRs were divided into four groups: (1) low bradycardic baroreflex (LB) where the baroreflex gain (BG) was between 0 and –1 bpm/mmHg with PHE; (2) high bradycardic baroreflex (HB), where the BG was < –1 bpm/mmHg with PHE; (3) low tachycardic baroreflex (LT) where the BG was between 0 and 3 bpm/mmHg with SNP; (4) high tachycardic baroreflex (HT) where the BG was > 3 bpm/mmHg with SNP.

We noted that 36.8% of the rats presented with an increased bradycardic reflex, while 27.8% demonstrated an attenuated tachycardic reflex. No significant alterations were noted regarding the basal MAP and HR. There were significant differences in the baroreflex sensitivity between SHRs in the same laboratory. One should be careful when interpreting studies employing the SHR as a research model.

## Summary

Diverse factors are involved in the onset of hypertension and different animal models have been developed in the investigation of this disease. These are the renovascular model, the DOCA-salt model, the neurogenic hypertension model and the genetic model of hypertension in spontaneously hypertensive rats (SHR). The SHR is a suitable model to study the course of hypertension, as it shares certain similarities with human essential hypertension. These similarities include: a genetic predisposition to high blood pressure with no known aetiology, an increase in total peripheral vascular resistance without volume expansion, and similar responses to drug treatment.^1^

It has been described in humans that arterial baroreflex function is significantly related to the prognosis of acute myocardial infarction, arrhythmias, heart failure and stroke.^2^-^6^ These observations indicate that such patients with a lower baroreflex sensitivity exhibit shorter survival times. Recently, it was reported that arterial baroreflex function or sensitivity plays an important role in the pathogenesis and prognosis of hypertension, atherosclerosis, aconitine-induced arrhythmia and LPS-induced shock.^5^,^7^,^8^ Conditions such as ageing, hypertension and radiation therapy to the neck diminish both arterial compliance and baroreflex sensitivity, and increased vascular stiffness may be an important clue to possible impaired baroreflex sensitivity.

An important question for the basic science researcher in hypertension is: are all spontaneously hypertensive rats (SHRs) the same? In 1987, it was described that SHRs from two different laboratories demonstrated significant differences with regard to growth rate and blood pressure.^9^

Although it was previously documented that a portion of normotensive Sprague-Dawley rats exhibited lower baroreflex sensitivity than their peers,^6^,^10^ no studies have yet been published that address the issue of whether there are any differences in baroreflex sensitivity between other types of rat of the same strain. It was recently reported that age is an important factor regarding baroreflex development in the SHR.^11^

The purpose of this study was to compare the baroreflex sensitivity between juvenile SHRs from the same laboratory, in order to explore the possibility that there may be intra-strain differences in the baroreflex sensitivity. We analysed the following: the baroreflex gain, the bradycardic and tachycardic peak and the heart rate (HR) range – the difference between the bradycardic and the tachycardic peak. Based on pilot studies (data not published), we expected significant differences between SHR rats of the same strain and from the same laboratory.

## Methods

The experiments were performed on eight-week-old SHRs from the same laboratory. Rats were housed individually in plastic cages under standard laboratory conditions. They were kept under a 12-hour light/dark cycle (lights on at 06:30) and they had free access to food and water. Housing conditions and experimental procedures were approved by the Institution’s Animal Ethics Committee. The minimum number of animals was used.

One day before the experiment, the rats were anaesthetised with ketamine (50 mg/kg i.p.) and xylaxine (50 mg/kg i.m.) and a catheter was inserted into the abdominal aorta through the femoral artery. This was done for the purpose of blood pressure and heart rate recording. The catheters were made of 4-cm segments of PE-10 polyethylene (Clay Adams, USA), heatbound to a 13-cm segment of PE-50. The catheters were tunneled under the skin and exteriorised at the animal’s dorsum.^11^

Approximately 24 hours after surgery the animals were kept in individual cages used in the transport to the experimental room. Animals were allowed 20 min to adapt to the conditions of the experimental room, such as sound and illumination, before starting blood pressure and heart rate recording. The experimental room was acoustically isolated and had constant background noise produced by an air exhauster. At least another 15-min period was allowed before beginning the experiment.

Pulsatile arterial pressure (PAP) of the freely moving animals was recorded using an HP-7754A pre-amplifier (Hewlett Packard, USA) and an acquisition board (model Powerlab 16SP, ADInstruments, Colorado Springs, CO, USA) connected to a computer. Mean arterial pressure (MAP) and heart rate (HR) values were derived from the PAP recordings and processed on-line.^11^

The baroreflex was tested with a pressor dose of phenylephrine (PE bolus: 8 μg/kg i.v.; Sigma Chemical) and a depressor dose of sodium nitroprusside (SNP bolus: 50 μg/kg i.v.; Sigma Chemical). The baroreflex was calculated as the derivation of HR as a function of the MAP variation (ΔHR/ΔMAP). There was an interval of at least 15 minutes between the infusions to allow the recovery of basal values. We also measured the bradycardic and tachycardic peak and the HR range – the difference between the bradycardic and tachycardic peak.^11^

We divided the rats into groups according to the baroreflex gain (BG): (1) low bradycardic baroreflex (LB) group: BG between 0 and –1 bpm/mmHg tested with PE; (2) high bradycardic baroreflex (HB) group: BG < –1 bpm/mmHg tested with PE; (3) low tachycardic baroreflex group (LT): BG between 0 and 3 bpm/mmHg tested with SNP; and (4) high tachycardic baroreflex group (HT): BG > 3 bpm/mmHg tested with SNP.

We compared the LB group with the HB group and the LT group with the HT group. We defined the values for bradycardic and tachycardic baroreflex gain according to a previous study.^12^

## Statistical analysis

Values are reported as the means ± standard error of means (SEM). HR, MAP, ΔHR, ΔMAP, bradycardic and tachycardic peak, HR range and ΔHR/ΔMAP were compared between HB and LB groups, as well as between HT and LT groups. After the distributions were evaluated with the Kolmogorov normality test, the unpaired Student’s *t*-test was used to verify differences between normal distributions, and the Mann-Whitney test was applied to assess differences between non-parametric distributions. Differences were considered significant when the probability of a type I error was less than 5% (*p* < 0.05).

## Results

Among all the 19 SHRs evaluated (based on baroreflex gain tested with PHE), approximately 37% presented with a higher parasympathetic baroreflex gain (HB group: < –1 bpm/mmHg). The majority of the animals who received PHE demonstrated lower baroreflex gain (LB group: between 0 and –1 bpm/mmHg).

In order to investigate the possibility that another cardiovascular parameter may have differed between the LB and HB groups, we compared baseline MAP and HR, the bradycardic and tachycardic peak, the HR range and baroreflex gain, tested with both PHE and SNP. No significant differences were noted between the two groups regarding the basal MAP and HR, the bradycardic peak and the sympathetic component of baroreflex gain (Table 1). However, there were significant differences in relation to the HR range, the tachycardic peak and the parasympathetic component of baroreflex gain.

**Table 1.** Baseline Level Of Mean Arterial Pressure (MAP) And Heart Rate (HR), Bradycardic And Tachycardic Peak, HR Range And Baroreflex Gain (BG) In HB (*n* = 7) And LB (*n* = 12) Groups. Mean ± SEM

*Variable*	*Group 1*	*Group 2*	p*-value*
MAP (mmHg)	166.14 ± 4.3	161 ± 3.5	0.3736
HR (bpm)	372.3 ± 12.8	337 ± 10.6	0.0527
Bradycardic peak (bpm)	319.6 ± 17.13	309.92 ± 11.6	0.6355
Tachycardic peak (bpm)	519.7 ± 11.7	471.1 ± 9.2	0.0.0048
HR range (bpm)	218.14 ± 18.4	162.45 ± 15.7	162.45 ± 15.7
BG (bpm.mmHg^-1^) PHE	–1.25 ± 0.09	–0.61 ± 0.064	< 0.0001
BG (bpm.mmHg^-1^) SNP	–1.94 ± 0.31	–2.87 ± 0.34	0.0817

**Fig. 1.**
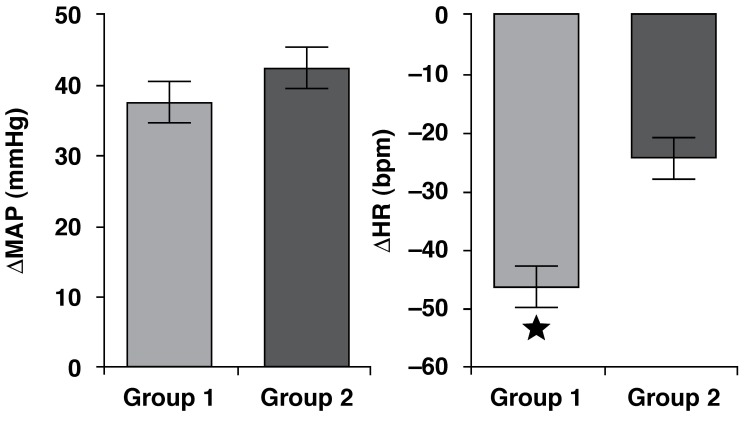
Increase in mean arterial pressure (MAP, mmHg) and decrease in heart rate (HR, bpm) in response to phenylephrine (PHE, 8 µg/kg i.v.) in HB (*n* = 7) and LB (*n* = 12) groups. *p < 0.0005: different from LB. Mean ± SEM.

PHE-induced increases in the MAP did not differ between the HB and LB groups (*p* = 0.33). However, bradycardic reflex responses to intravenous PHE were significantly decreased in the LB group (*p* = 0.0001) (Fig. 1).

**Fig. 2.**
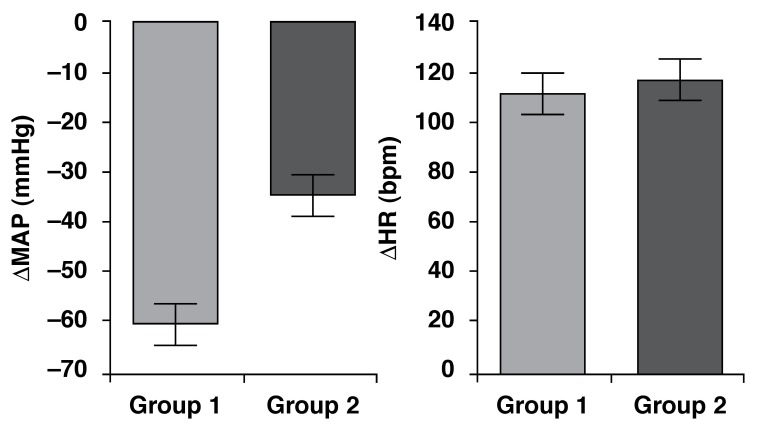
Decrease in mean arterial pressure (MAP, mmHg) and decrease in heart rate (HR, bpm) in response to sodium nitroprusside (SNP, 50 µg/kg i.v.) in HB (*n* = 7) and LB (*n* = 12) groups. Mean ± SEM.

We also compared SNP-induced decreases in MAP and the tachycardic response to i.v. SNP between the HB and LB groups. MAP decreases in response to SNP tended to be reduced in the LB group (*p* = 0.0709), however, they did not reach statistical significance. With regard to the tachycardic reflex response, we did not note significant differences between the two groups (*p* = 0.7229) (Fig. 2).

When baroreflex gain was tested with SNP, we noted that among all the 19 SHRs analysed, approximately 27% presented with a higher baroreflex gain (HT group: > 3 bpm/mmHg), while the majority (approximately 73%) presented with a lower baroreflex gain (LT group: between 0 and 3 bpm/mmHg).

We observed significant differences with regard to the sympathetic component of the baroreflex gain (Table 2). There were no significant differences between the two groups regarding basal MAP and HR, the bradycardic and tachycardic peak, HR range or the parasympathetic component of the baroreflex gain.

**Table 2.** Baseline Level Of Mean Arterial Pressure (MAP) And Heart Rate (HR), Bradycardic And Tachycardic Peak, HR Range And Baroreflex Gain (BG) LT (*n* = 13) And HT (*n* = 5) Groups. Mean ± SEM

*Variable*	*Group 3*	*Group 4*	p*-value*
MAP (mmHg)	163.3 ± 3.42	158.4 ± 3.59	0.4278
HR (bpm)	352.15 ± 11.5	347 ± 17.3	0.821
Bradycardic peak (bpm)	314.15 ± 12.5	309.6 ± 17.17	0.8448
Tachycardic peak (bpm)	486.5 ± 10.2	499 ± 20.6	0.5548
HR range (bpm)	182.1 ± 16.2	89.4 ± 25.97	0.8143
BG (bpm.mmHg^-1^) PHE	–0.91 ± 0.12	–0.68 ± 0.09	0.2812
BG (bpm.mmHg^-1^) SNP	–1.96 ± 0.16	–3.93 ± 0.4	< 0.0001

**Fig. 3.**
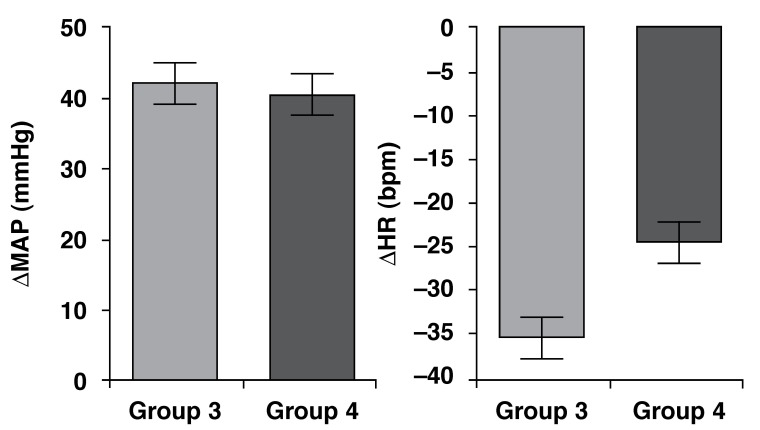
Increase in mean arterial pressure (MAP, mmHg) and decrease in heart rate (HR, bpm) in response to phenylephrine (PHE, 8 µg/kg i.v.) in LT (*n* = 13) and HT (*n* = 5) groups. Mean ± SEM.

When comparing the HT and LT groups with regard to PHE-induced increases in MAP, no statistically significant differences were observed (*p* = 0.7384). Furthermore, the bradycardic reflex responses to increases in arterial pressure were not different between the two groups (*p* = 0.161) (Fig. 3).

**Fig. 4.**
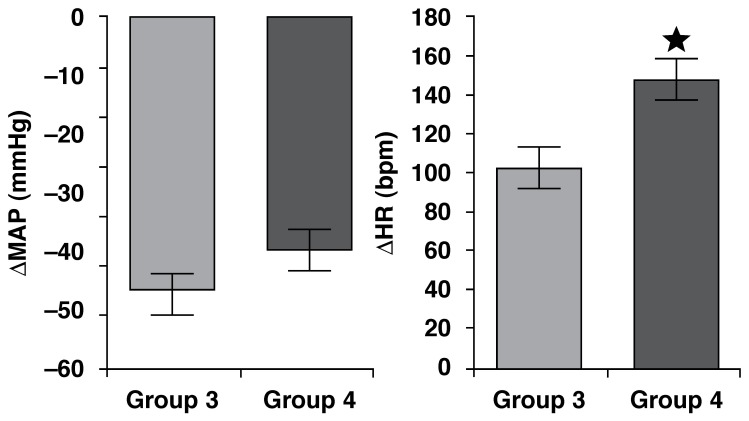
Decrease in mean arterial pressure (MAP, mmHg) and decrease in heart rate (HR, bpm) in response to sodium nitroprusside (SNP, 50 µg/kg i.v.) in LT (*n* = 13) and HT (*n* = 5) groups. *p < 0.005: different from LT group. Mean ± SEM.

Decreases in MAP in response to SNP were similar between the HT and LT groups (*p* = 0.6706) (Fig. 4). However, tachycardic reflex responses to decreases in arterial pressure were significantly reduced in the LT group (*p* = 0.0044).

## Discussion

In this study we compared the baroreflex sensitivity between juvenile SHRs by bolus infusions of the vasopressor PHE and the vasodepressor SNP. Our findings demonstrate that SHRs from the same laboratory can be divided into two groups, based on baroreflex gain (HB vs LB groups, due to parasympathetic responses, and LT vs HT groups, due to sympathetic responses).

We compared these groups and found that the parasympathetic component of the baroreflex gain and the bradycardic reflex response to increases in arterial pressure were significantly reduced in approximately 63% of the rats investigated. We also found that the sympathetic component of the baroreflex gain and the tachycardic reflex in response to reductions in blood pressure were significantly reduced in approximately 73% of the rats studied. Considering that no ketamine effect remains in rats after 24 hours with regard to baroreflex tests,^13^ we discarded the possibility of any interference of this drug in our study.

In this study, baroreflex function was examined by bolus infusions of vasopressors and depressors and we measured HR changes in response to arterial pressure increases or decreases, caused by the intravenous infusion of SNP or PHE, respectively. According to our findings, approximately 63% of 19 SHRs (LB group) presented with reduced bradycardic reflex responses to increases in arterial pressure and decreased baroreflex gain, tested with the α_1_-adrenergic agonist PHE. Baseline HR tended to be reduced in the LB group. However, it did not reach statistical significance (*p* = 0.0527). Furthermore, the LB group also demonstrated a reduced tachycardic peak and HR range. This was probably due to a lower plateau of HR (maximal bradycardic response) as well as a higher plateau of HR (maximal tachycardic response), although the bradycardic peak was not statistically significantly different.

We concluded that approximately one in four SHRs demonstrated a significant increase in reflex tachycardic gain. Much attention has been focused on the role of sympathetic activity regarding the onset of hypertension in the SHR. Previous research has shown an elevation of sympathetic drive to blood vessels in awake SHRs and has suggested that this is important in the maintenance of increased blood pressure.^14^,^15^ It is possible that this elevation in sympathetic output is not primarily a consequence of changes in either the baroreceptor reflex^15^ or chemoreflex function, but rather, is a product of a modification of the central neural circuitry involved in generating the sympathetic output.^16^

In view of the above considerations, although there were no significant alterations with regard to basal MAP and HR between the HT and LT groups, we may not discard the possibility that rats with an increased tachycardic reflex may be more susceptible to higher sympathetic nervous activity, since we did not measure this. Future studies are necessary to explore this possibility.

The currently reported differences in baroreflex sensitivity between SHRs from the same laboratory might be due to factors such as spontaneous mutations, genetic contamination of the breeding stock, or non-genetic influences (e.g. vertically transmitted diseases or differences in the prenatal and neonatal environments existing at different breeding facilities).^17^ In the article that describes the initial development of the SHR, Okamoto and Aoki18 stated that the rats were selected from a Wistar strain that had been maintained by inbreeding. Therefore, it is conceivable that the normotensive Wistar rats sent from Kyoto to the National Institute of Health (NIH) in 1971 were at least partially inbred. However, the precise circumstances of the brother–sister mating are not clear because records from the NIH indicate that: (1) the SHR were developed from an ‘outbred Wistar Kyoto male’ and (2) the Wistar rats from Kyoto used by the NIH to breed WKY were from ‘non-inbred’ stock.^18^

In this study baroreflex function was evaluated in conscious rats, since baroreflex activity is blunted under anesthesia,^19^,^20^ thus reducing the range of HR, which would impact on the outcome in an analysis on a restricted portion of the baroreflex response. Therefore, we believe that this study provides accurate information regarding the discrepancy of baroreflex function between rats of the same strain (in our case the SHR strain). It would also be interesting to compare other cardiovascular reflexes (such as the cardiopulmonary reflex and chemoreflex) in other strains of rat, such as the SHR stroke prone (SHRSP), and in other animals, such as rabbits and mice.

These data present clinically relevant information, since the baroreceptor reflex is currently studied mainly in different models and strains of rats, aiming to prevent hypertension development in the human,^11^,^20^,^21^ due the fact that reduced baroreflex function is indicative of cardiovascular disease.^22^-^24^ Since SHR strains are being used extensively throughout the world, researchers should be aware of the genealogical background of the SHR.

It was also previously shown that genetic markers of WKY, such as asylosterase isozyme patterns, differed among the available strains of WKY (unpublished observation). Such information is useful for researchers who are using SHRs in comparison with WKY, and may assist in understanding the correct usage of SHRs, as well as the control WKY strain.

## Conclusion

We demonstrated a significant variation in the baroreflex sensitivity between SHRs of the same laboratory and we concluded that this may significantly influence future studies employing the SHR as research model.
